# Linking N_2_O emission from biochar-amended composting process to the abundance of denitrify (*nirK* and *nosZ*) bacteria community

**DOI:** 10.1186/s13568-016-0208-x

**Published:** 2016-05-20

**Authors:** Shuqing Li, Lina Song, Yaguo Jin, Shuwei Liu, Qirong Shen, Jianwen Zou

**Affiliations:** Jiangsu Key Laboratory of Low Carbon Agriculture and GHGs Mitigation, College of Resources and Environmental Sciences, Nanjing Agricultural University, Nanjing, 210095 China; Jiangsu Key Lab and Engineering Center for Solid Organic Waste Utilization, Jiangsu Collaborative Innovation Center for Solid Organic Waste Resource Utilization, Nanjing Agricultural University, Nanjing, 210095 China

**Keywords:** Biochar, Nitrous oxide, Denitrifying genes abundance, Manure composting, Modeling

## Abstract

Manure composting has been recognized as an important anthropogenic source of nitrous oxide (N_2_O) contributing to global warming. However, biochar effect on N_2_O emissions from manure composting is rarely evaluated, especially by linking it to abundance of denitrifying bacteria community. Results of this study indicated that biochar amendment significantly reduced N_2_O emissions from manure composting, primarily due to suppression of the *nirK* gene abundance of denitrifying bacteria. Pearson’s correlation analysis showed a significant positive correlation between *nirK* abundance and N_2_O fluxes, while a negative correlation between *nosZ* density and N_2_O fluxes. Simultaneously, a linear correlation between *nirK* gene abundance minus *nosZ* gene abundance with N_2_O fluxes was also observed. In addition, a statistical model for estimating N_2_O emissions based on the bacterial denitrifying functional genes was developed and verified to adequately fit the observed emissions. Our results highlighted that biochar amendment would be an alternative strategy for mitigating N_2_O emissions during manure composting, and the information of related functional bacterial communities could be helpful for understanding the mechanism of N_2_O emissions.

## Introduction

Nitrous oxide (N_2_O) is an important long-lived potent greenhouse gas contributing to current climate change, with 265 times greater global warming potential than that of carbon dioxide (CO_2_) on a mass basis over the 100-year time horizon (IPCC 2013). Manure composting as an important management strategy for sustainable use of livestock could reduce treatment costs but generate organic fertilizer for improvement of soil fertility (Larney and Hao 2007). However, manure composting leads to a large amount of nitrogen loss released as N_2_O or NH_3_ (Maeda et al. 2011). The annual global N_2_O emissions derived from manure composting were estimated to be 1.2 × 10^6^ metric tons (Czepiel et al. 1996), which accounts for approximately 30–50 % of the annual global total of agricultural N_2_O emissions in most countries (Chadwick et al. 2011). Therefore, it is of great concern on developing effective alternatives for reducing N_2_O emissions and nitrogen losses during manure composting.

Recently, the importance of microbial traits involved in N_2_O production processes has gained worldwide concern (e.g., Maeda et al. 2011). It has been reported that N_2_O emissions from manure composting mainly occurred at the cooling stages of composting during denitrification driven by bacteria (Maeda et al. 2011), i.e., NO_3_^−^, NO_2_^−^, NO, and N_2_O are sequentially reduced by the catalyzation of nitrate reductase (*narG*), nitrite reductase encoding (*nirS*/*nirK*), nitric oxide reductase (*norB*), and nitrous oxide reductase (*nosZ*, functional for yielding N_2_), respectively (Maeda et al. 2011; Wang et al. 2013). Therefore, N_2_O emissions during manure composting are the result of net balance between its production (NO_3_^−^ → N_2_O) and consumption (N_2_O → N_2_) (Maeda et al. 2011). Several previous studies have investigated the dynamics of these functional genes abundance involved during manure composting processes under different conditions, and highlighted their interplay with observed N_2_O emissions and physicochemical characteristics (Angnes et al. 2013; Wang et al. 2013). Unfortunately, although the microbial pathways involved in N_2_O formation have been well documented, most of the established statistical models for estimating N_2_O emissions were currently still limited based on physicochemical parameters but without taking the microbial traits into consideration (Hu et al. 2015).

To date, various options have been reported for mitigating N_2_O emissions from manure composting, including the composting parameters modulation (e.g., C/N ratio, water content, pH), N_2_-fixing bacteria inoculation, and material amendments (Dias et al. 2010; Ogunwande et al. 2008; Pepe et al. 2013; Wang et al. 2013). Recently, biochar amendment has been increasingly encouraged as a potential approach for reducing N_2_O emissions from both manure composting systems and soils (Kammann et al. 2012; Wang et al. 2013). As a carbon-rich material derived by slow pyrolysis of biomass, biochar amendment can help to improve the NH_3_/NH_4_^+^ retention during the composting process (Steiner et al. 2010). Wang et al. (2013) examined the dynamics of functional microbial community during the windrow composting process, and revealed a correlation between the denitrifying bacteria and N_2_O emissions, but lack of a systematic link using the modeling approach.

In this study, an in situ measurement of N_2_O fluxes as regulated by biochar amendment over the whole life-cycle of manure windrow composting was taken to address the following concerns: (1) to evaluate the role of biochar in regulating N_2_O emissions during the composting process; (2) to give an insight into the interplay between N_2_O emissions and the abundance of relevant functional genes involved in denitrification; (3) and to develop a statistical model for estimating N_2_O emissions by integrating both the bacterial functional genes and physiochemical parameters. The results of this study would help to advance our knowledge on the potential effects of biochar for mitigating N_2_O emissions during manure composting, and simultaneously establish a link between N_2_O fluxes and the information of related functional bacterial communities.

## Materials and methods

### Experimental design

The windrow composting experiment was initiated on December 24, 2013 in a commercial organic fertilizer company (Jiangyin Lianye Biological Science and Technology Co., Ltd), located in Jiangsu Province, China. The total composting period was lasted for 64 days, and two types of composts were carried out: CK and Biochar. Each treatment was set up with three replicated composting piles. Each plie was sized as 12 m (length) × 2.8 m (width) × 1 m (height). Before the piles were constructed, compost feedstocks, including cattle manure and rice-chaff, were mixed in a ratio of 75:25 % (v/v) on a fresh weight basis. The cattle manure and rice straw were obtained from a cattle ranch and local paddy rice fields, respectively. The piles of biochar were received an additional biochar amendment with 3 % (w/v). Biochar used in this study was produced from wheat straw at a temperature of approximately 450 °C from a local company. Physicochemical properties of biochar were listed as follows: a total C content of 467.0 g kg^−1^, a total N content of 5.6 g kg^−1^, a pH of 9.4 (1:2.5 H_2_O), cation exchange capacity of 24.1 cmol kg^−1^ and ash content of 20.8 %.

The composting process can be generally divided into two phases. The phase I was the bio-oxidative phase that mechanical turning was taken once every 2 days for 24 days (December 24, 2013 to January 16, 2014). Thereafter, the piles were moved to the aside place for post-maturation during the phase II. The phase II was the cooling and maturing phase without pile turning for 40 days (January 17, 2014 to February 26, 2014) (Bernal et al. 2009; Chen et al. 2014).

### Measurement of N_2_O fluxes

Besides that gas samples were collected regularly once a week over the whole composting process, supplementary gas sampling episodes were occasionally taken as needed to capture high flux peaks for the two pile treatments. The N_2_O emission was simultaneously measured using a modified vented chamber method (Chen et al. 2014; Hou et al. 2001; Mosier and Hutchinson 1981; Zou et al. 2005). Before sampling, PVC chamber bases (30 cm length × 30 cm width × 25 cm height) were pre-inserted 25 cm into the peak of piles to reduce the disturbance. When gas sampling, the opaque chamber (30 cm length × 30 cm width × 50 cm height) was placed on the bases and the bottom edge was sealed by water. At 0, 5, 10, 20, and 30 min after chamber closure, gas samples were extracted using 60 mL plastic syringes and immediately injected into 50 mL pre-evacuated Exetainer (Chen et al. 2014; Hou et al. 2001).

The N_2_O concentration was determined using the gas chromatograph method (Zou et al. 2005), which was performed with a modified gas chromatograph (Agilent 7890, Agilent Technologies) equipped with an electron capture detector (ECD) (Liu et al. 2012; Zou et al. 2005). Each pile along its length was sub-divided into three sections that were treated as three parallel locations to minimize spatial heterogeneity of gas and compost sampling. The N_2_O fluxes were calculated by a non-linear approach and the mean of fluxes taken from three parallel sections within each windrow represent flux measurement of the sampling windrows. Average fluxes and standard deviations of N_2_O were calculated from three replicated windrows. The cumulative N_2_O emissions were sequentially accumulated from the emissions between every two adjacent intervals of the measurements (Zou et al. 2005).

### Physicochemical parameters determination

Windrow temperature at 30 cm depth of piles was measured using a mercury thermometer on each gas sampling day. To examine dynamics of physiochemical parameters and functional microbial abundance, the compost samples were randomly collected from three longitudinal locations from different parts of piles. The collected samples were divided into three parts. Two parts were immediately preserved at 4 °C or −80 °C, while the other part was air-dried, sieved, and stored for further analysis. The moisture content of different fresh samples was determined based on the weight loss by oven-drying at 105 °C. To analyze the water-soluble fractions of the compost material, the mixture of 20 g fresh compost samples with 200 mL deionized water (1:10 w/v ratio) was shaken on a horizontal shaker at 25 °C (Castaldi et al. 2008). To determine the NH_4_^+^ and NO_3_^−^ concentration of compost samples, 5 g of fresh samples were extracted with 100 mL 2 M KCl solution (1:20 w/v ratio) at room temperature. The solutions were measured using the three wavelength ultraviolet spectrometry by an ultraviolet spectrophotometer (HITACHI, U-2900, Japan).

### DNA extraction

DNA was extracted from the compost samples using the Ultraclean soil DNA isolation kit (MoBio, USA), as described in the manufacturer’s instructions. Each DNA sample for next-analysis was the mixture of three independent DNA extractions from one compost sample. The DNA sample concentration was determined by a Nanodrop (Thermo Scientific, USA).

### Real-time q-PCR assay

Real-time q-PCR assays were performed for investigation of the functional microbial community dynamics during the composting process. 16S rRNA and two genes, encoding the key enzymes involved in N_2_O emission, nitrite reductase (*nirK*) and nitrous oxide reductase (*nosZ*), were amplified using SYBR^®^ Premix ExTaq™ kit (Takara, Dalian). The sequences of the primers used were referenced in Table 1. For the standard curve preparation, the PCR amplified fragments for three genes were cloned into pMD 18-T vector and sequenced.

**Table 1:** The primers used for quantitative PCR in this study

Gene	Name	Sequence (5′–3′)	Thermal profile	No. cycles	Product size (bp)	Reference
*nirK*	*nirK*F1aCu	ATCATGGTSCTGCCGCG	30 s-95 °C, 95 °C-15 s, 55 °C-30 s, 72 °C-30 s, 80 °C-30 s	1	473	Henry et al. (2004)
	*nirK*R3Cu	GCCTCGATCAGRTTGTGGTT	95 °C-5 s, 58 °C-34 s, 72 °C-15 s 95 °C-15 s, 55 °C-30 s, 72 °C-30 s, 80 °C-30 s	40		
*nosZ*	*nosZ*-F	AGAACGACCAGCTGATCGACA	30 s-95 °C, s, 80 °C-30 s	1	300	Scala and Kerkhof (1998)
	*nosZ*-R	TCCATGGTGACGCCGTGGTTG	95 °C-5 s, 60 °C-34 s, 72 °C-15 s 95 °C-15 s, 55 °C-30 s, 72 °C-30 s, 80 °C-30 s	40		
*16S rRNA*	515F	GTGCCAGCMGCCGCGG	30 s-95 °C,	1	392	Zhou et al. (2011)
	907R	CCGTCAATTCMTTTRAGTTT	95 °C-5 s, 55 °C-34 s, 72 °C-15 s 95 °C-15 s, 55 °C-30 s,72 °C-30 s, 80 °C-30 s	40		

*M* A/C, *R* A/G

The q-PCR assays was carried out in 20 μL reaction volume containing 10 μL SYBR Premix ExTaq, 0.4 μL each primer (10 μmol^−1^), 0.4 μL ROX reference dye II (50×), 2 μL template DNA and 6.8 μL sterlized water. Reactions were perfomed triplicate using 7500 system (Appled Biosytem, USA). The information of primers and q-PCR reaction process was listed in Table 1. Target gene copy numbers in compost samples were calculated from the standard curves and dry weight of compost samples.

### Statistics

All data were reported as means and standard deviations. The pairwise correlation was conducted for the correlation between N_2_O fluxes, functional gene abundance (*nirK* and *nosZ*), and related physiochemical parameters (temperature, moisture, NH_4_^+^, NO_3_^−^). A linear model with the personality of ordinary least squares (OLS) was used to fit the N_2_O fluxes by physiochemical parameters and related functional gene abundance. All statistical analyses were performed using JMP version 9.0 (SAS Institute, USA, 2010).

## Results

### N_2_O fluxes

**Fig. 1:**
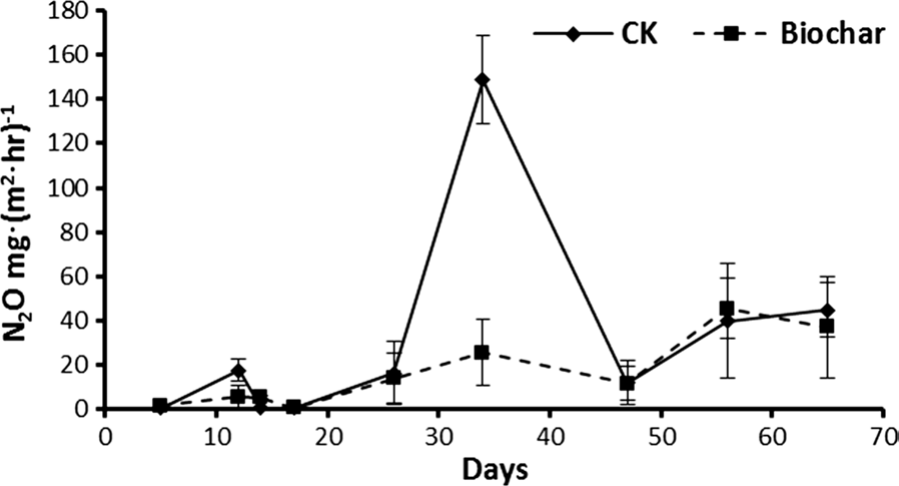
Changes in N_2_O emission rate during the windrow composting (mean ± 1 SD)

**Fig. 2:**
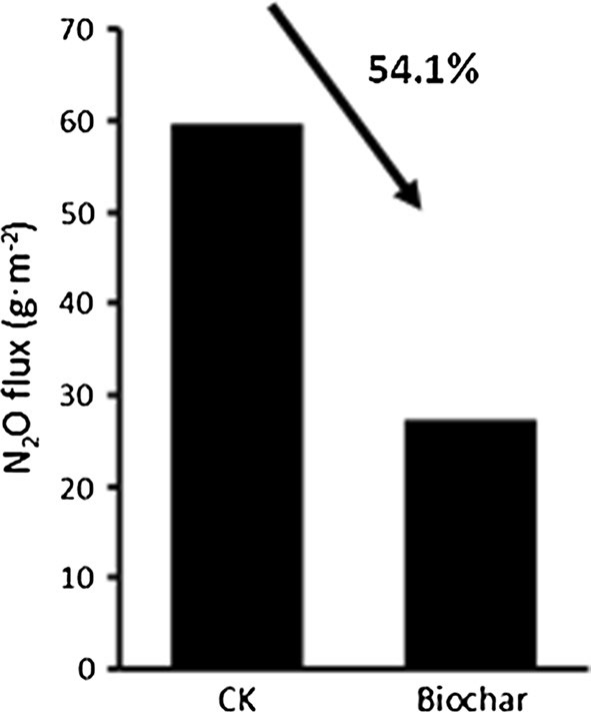
The cumulative N_2_O emissions during the 65-day period of composting

The fluxes of N_2_O were low during the early composting stage in both piles and got raised since 26 days. A substantially higher N_2_O emission peak was captured in the control pile on day 34 as compared with that in the biochar-amended pile (148.65 vs. 25.56 mg m^2^ h^−1^). Thereafter, N_2_O fluxes were similar both the two treatments, ranging from 10.00 to 45.00 mg m^2^ h^−1^ until the end of experiment (Fig. 1). Over the 65-day compositing period, biochar amendment significantly decreased the cumulative N_2_O emissions by 54.1 % relative to the controls (Fig. 2).

### Physiochemical parameters

**Fig. 3:**
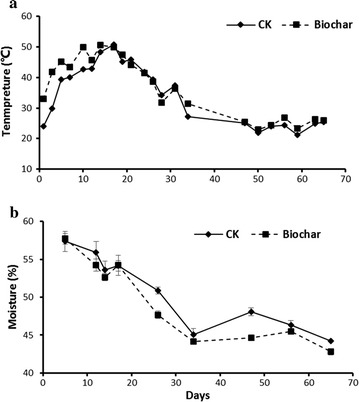
Changes in temperature (**a**) and moisture (**b**) of composting materials during the windrow composting process

On average, biochar amendment increased the pile temperature as compared with control pile over the total observation cycle (Fig. 3a). Peak of the composting temperature in the biochar-amended piles was observed on day 14 (approximately 50.6 °C) and was kept for 6 days, while the temperature of control piles reached 50.7 °C on day 17 but rapidly levelled off. Afterwards, the temperature of both pile types gradually decreased to below 30 °C and then remained stable till the end (Fig. 3a). Significant decreases in water content were observed during the composting process for both piles, and biochar addition led to relatively lower water content as compared to the control pile over the observation cycle (Fig. 3b).

Obvious decrease of NH_4_^+^ and corresponding increase of NO_3_^−^ occurred at the maturation stages in both piles (after 47 days). The highest concentration of NO_3_^−^ was relatively lower in biochar-amended piles than in the control piles (541.83 vs. 593.70 mg kg^−1^, day 56, Table 2), but their difference was not statistically significant (*p* > 0.05).

**Table 2:** The concentration of NH_4_^+^ and NO_3_^−^ during the composting process

Composting time (days)	NH_4_^+^(mg/kg)		NO_3_^−^ (mg/kg)	
	CK	Biochar	CK	Biochar
5	1026.45 ± 156.30	1150.39 ± 123.72	143.08 ± 21.95	151.68 ± 16.33
12	608.99 ± 89.77	552.07 ± 89.23	179.55 ± 17.40	205.35 ± 36.72
14	547.95 ± 101.23	580.66 ± 78.01	182.57 ± 29.80	192.59 ± 9.70
17	568.46 ± 45.89	552.64 ± 46.93	157.66 ± 31.90	175.10 ± 23.89
26	558.96 ± 56.11	580.70 ± 23.43	141.73 ± 10.91	168.62 ± 30.01
34	498.31 ± 30.03	573.46 ± 92.10	247.79 ± 27.18	177.87 ± 29.55
47	415.75 ± 56.30	544.38 ± 64.02	345.26 ± 38.45	218.10 ± 48.74
56	113.93 ± 19.78	212.17 ± 59.30	593.70 ± 68.29	541.83 ± 83.29
65	125.02 ± 34.04	288.12 ± 32.91	591.53 ± 56.48	503.60 ± 76.20

Data was presented as mean ± standard error

### Abundance dynamics of denitrifying bacteria community

**Fig. 4:**
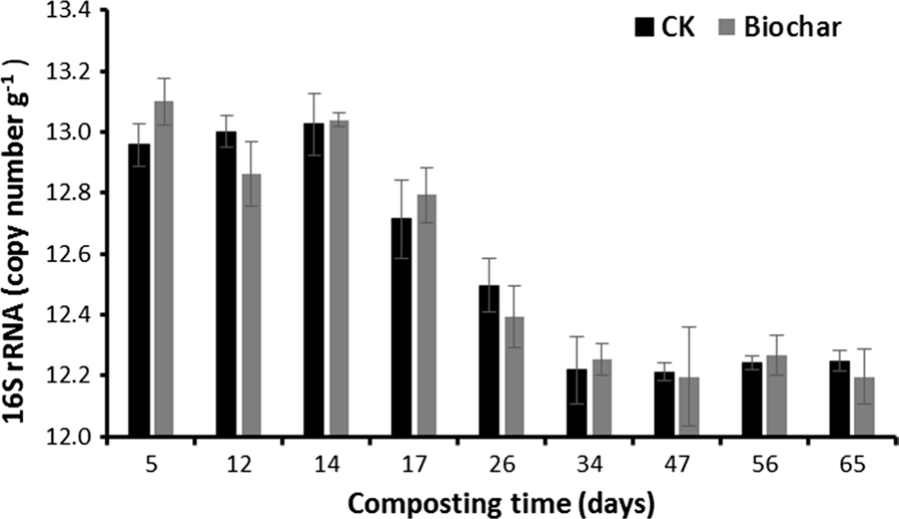
Changes in gene copy numbers per gram of compost (dry matter) for 16S rRNA. *Error bars* indicate standard error of the mean (SE) of triplicate q-PCR reactions

Similar patterns of the copy numbers of total bacterial 16S rRNA during the composting process were observed between the two pile treatments, suggesting biochar amendment did not significantly alter the whole bacterial density (Fig. 4). Specifically, the abundance remained high but stable during the thermophilic stage (day 5–14) and then substantially decreased after the temperature reaching the peak on day 34. Subsequently, the population abundance remained at low level until the end of experiment (Fig. 4).

**Fig. 5:**
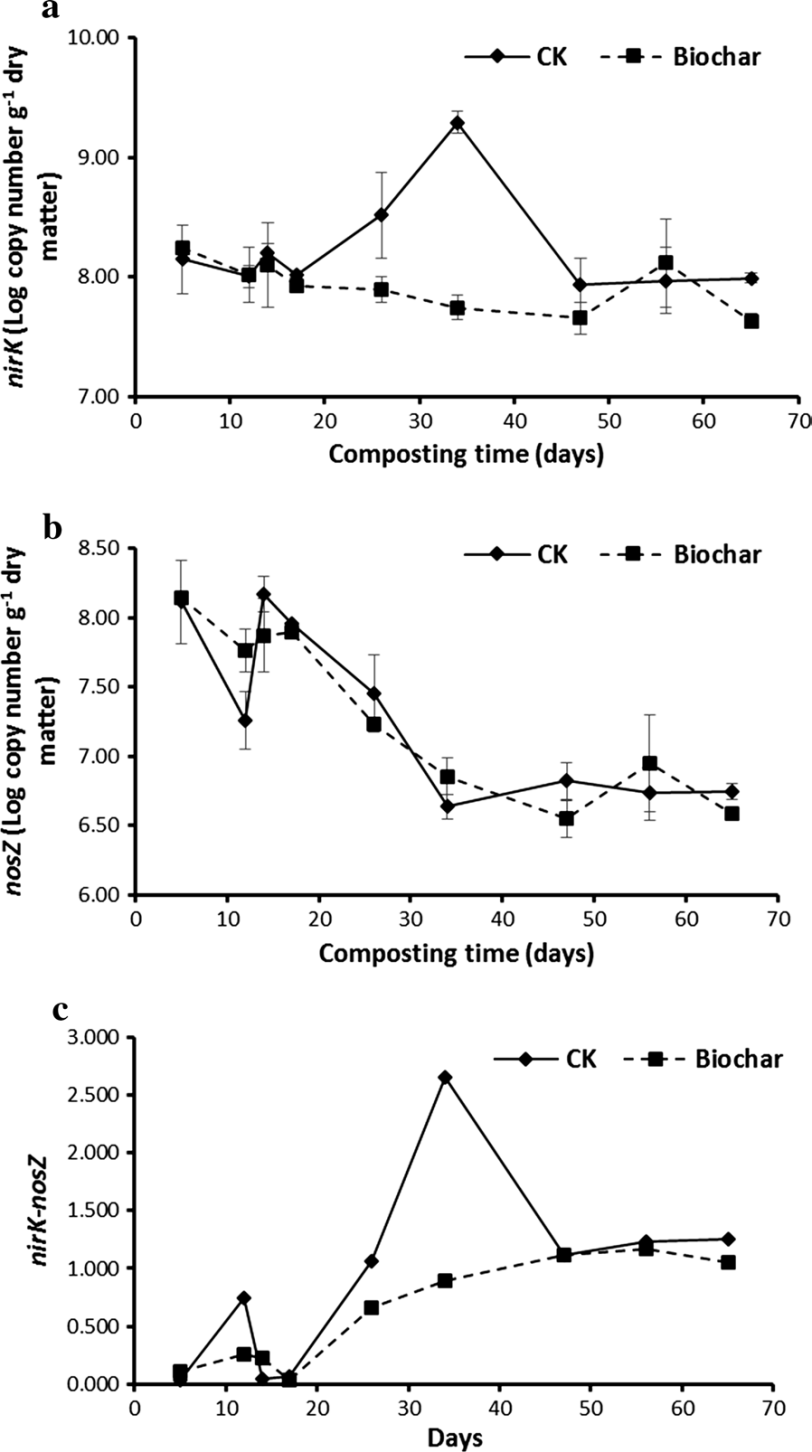
Dynamics of population of *nirK* (**a**) and *nosZ* (**b**) and the *nirK* gene abundance minus *nosZ* gene abundance (**c**) during the windrow composting process. *Error bars* indicate standard error of the mean (SE)

In the control piles, the *nirK* abundance was gradually increased and attained its peak of 9.29 log copy numbers·g^−1^ on day 34, corresponding to the peak of N_2_O fluxes at the same time (Fig. 5a). However, no such peaks occurred in biochar-amended piles, and population levels of *nirK* kept stable and relatively lower over the whole composting cycle, ranging from 7.64 to 8.25 log copy numbers·g^−1^ (Fig. 5a). Dynamic patterns of *nosZ* density were similar between the both pile treatments, showing a significant decrease trend from day 14 to day 34, and then remained stable around 6.70 log copy numbers·g^−1^ to the end of experiment (Fig. 5b). In contrast, the *nirK*-*nosZ* value showed an overall increase in both piles, and the mean values in control piles were greater than those in the biochar-added piles, especially the values on day 34 (1.40 in the control piles vs. 1.13 in the biochar-amendment piles, Fig. 5c). And then, the values in both pile treatments had a tendency to be uniform until the end of experiment.

### Correlations of N_2_O fluxes with gene abundance and physiochemical parameters

**Fig. 6:**
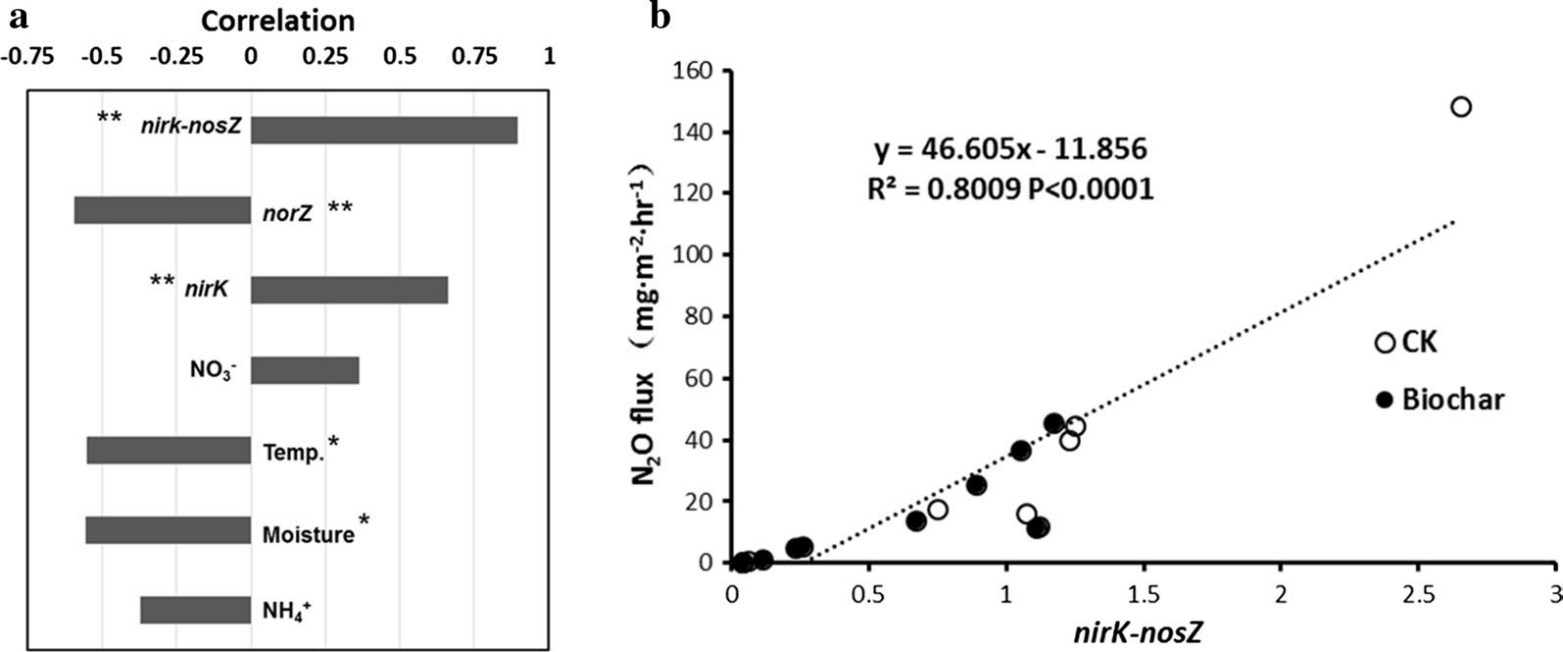
**a** Correlation analysis between N_2_O emission and physiochemical/microbial factors. **b** Simple regression analysis of N_2_O emission and the *nirK* gene abundance minus *nosZ* gene abundance

**Fig. 7:**
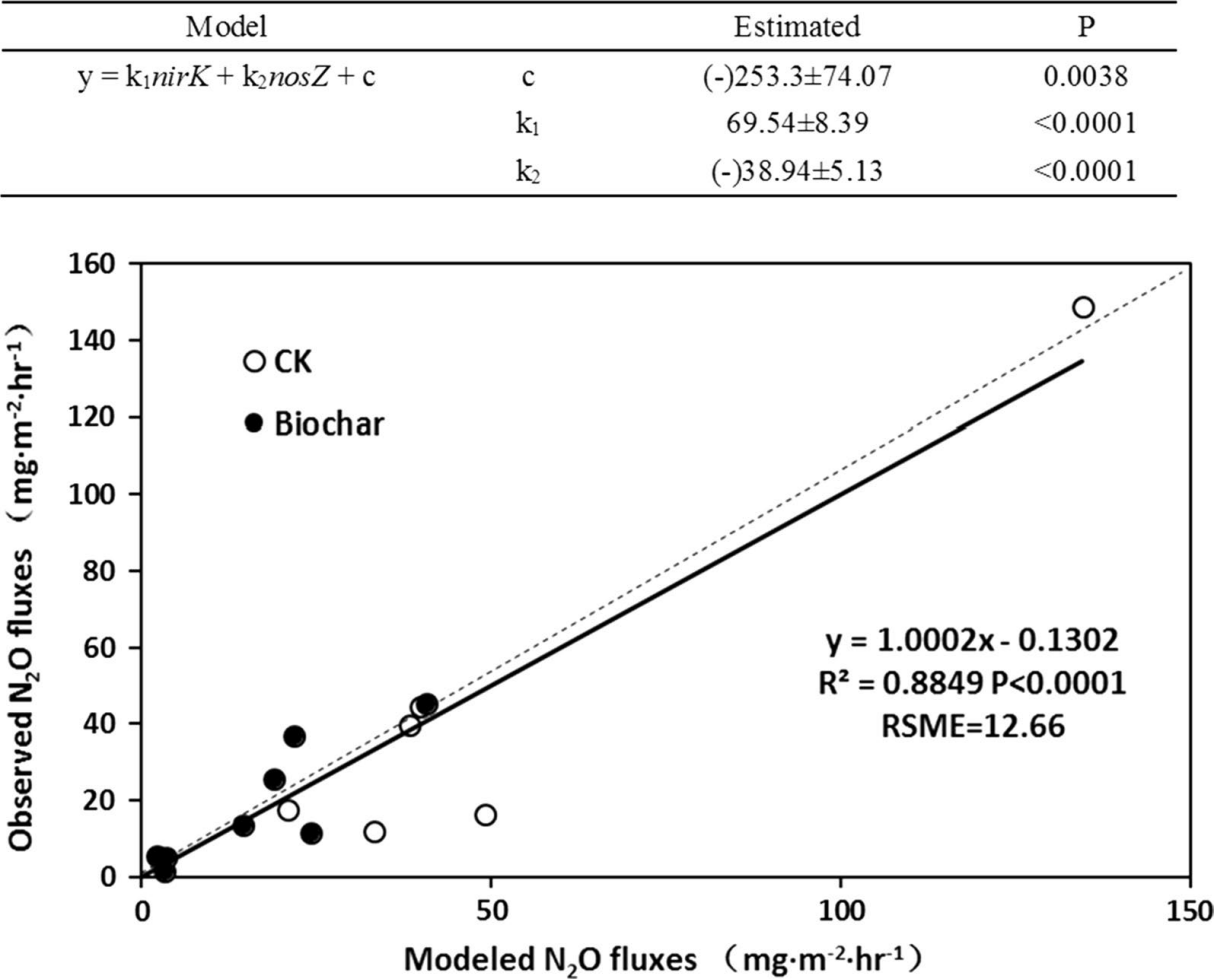
A schematic model for explaining the N_2_O fluxes dynamics associated with abundance of *nirK* and *nosZ*

For physiochemical parameters, the NO_3_^−^ content was positively correlated with N_2_O fluxes, while temperature, moisture, and NH_4_^+^ content showed a negative correlation with N_2_O fluxes (Fig. 6a). A significant positive correlation was observed between *nirK* abundance and N_2_O fluxes (*r*^2^ = 0.67, *p* < 0.01), while a negative correlation existed between *nosZ* density and N_2_O fluxes (*r*^2^ = 0.55, *p* < 0.01) (Fig. 6a). In particular, the linear regression analysis suggested a significant positive correlation between N_2_O fluxes and *nirK*-*nosZ* value (*r*^2^ = 0.80, *p* < 0.001) in the both pile types (Fig. 6b), which could also serve as a good predictor in regression model for stimulating N_2_O emissions from windrow composting systems. Thereby, we further developed a statistical model for explaining the dynamics of N_2_O fluxes by simultaneously taking abundance of both *nirK* and *nosZ* genes into account (Fig. 7). The significant correlation (*r*^2^ = 0.88) between the predicted and observed N_2_O fluxes suggested that the statistical model established based on the abundance of functional genes could be applied to estimate GHG emissions from windrow composting systems (Fig. 7).

## Discussion

As recognized as an importance source of CH_4_ and N_2_O, manure composting has gained extensive attention of developing available strategies for alleviating GHG emissions (Chadwick et al. 2011; Owen and Silver 2015; Tsutsui et al. 2013). In this study, primary N_2_O fluxes were observed at middle stage of the composting in both piles (after 26 days), mainly because of the decreased temperature and limited oxygen availability. The fluxes of N_2_O in this study have also been supported by numerous previous relevant studies (Sanchez-Monedero et al. 2010; Tsutsui et al. 2013; Wang et al. 2013). Importantly, biochar amendment effectively reduced the N_2_O fluxes during the manure composting by 54.1 % as compared with the control piles, especially after the cooling stage from 34 days after treatment (Figs. 1, 2), suggesting its potential of application in agricultural production for migrating GHG emissions.

Previous studies have highlighted the positive performance of biochar on reduction of N_2_O emissions in both composting and soil systems. The involved mechanisms have been summarized as physical absorption, improved soil aeration (Zhang et al. 2010), mediation of denitrifiers (Wang et al. 2013), repressing denitrification and inducing N_2_O-reductase activities (Yanai et al. 2007), and regulating of N transformations (Clough and Condron 2010). Recently, it has been recognized that the N_2_O emissions during manure composting were the balance between production (NO_3_^−^ → N_2_O, primarily catalyzed by nitrite reductase encoding by *nirS/nirK*) and consumption (N_2_O → N_2_, catalyzed by nitrite reductase encoding by *nosZ*) (Maeda et al. 2011). Since *nirK* has been suggested to be the dominant denitrification gene as compared with *nirS* in the composting system (Wang et al. 2013; Zhang et al. 2015), the abundance of *nirK* and *nosZ* was investigated for understanding the microbial mechanisms that involved in the biochar-mediated N_2_O mitigation. As expected, addition of biochar counteracted the significant raise of *nirK* abundance as compared with the control pile, especially on day 34 at which the N_2_O emissions reached peak in the control piles in contrast to much lower N_2_O fluxes in the biochar-amended piles (Figs. 1, 6a). Moreover, the population of *nosZ* was found to be similar in both piles (Fig. 6b), therefore it could be hypothesized that the N_2_O reduced by biochar amendment was mainly attributed to the alternation of bacterial gene abundance of *nirK*. Previous studies also highlighted that biochar application could lowered the abundance of *nirK* in manure composting (Wang et al. 2013), and *nirS* (or relative proportion) under field condition (Anderson et al. 2014; Bai et al. 2015).

There are several probable explanations for the effects of biochar amendment on denitrification gene abundance. First, improvement of soil aeration by biochar amendment due to its nano-porosity and large specific surface areas, as well as the consequent lower moisture content (Fig. 4), could influence the oxygen availability and redox condition, thereby depress the abundance, diversity, and activity of the denitrifiers (Wang et al. 2013; Zhang et al. 2010). Second, ethylene generated from biochar could inhibit the abundance and activity of soil microbiota (Spokas et al. 2010). Nevertheless, additional studies are highly needed to exploring the detailed response mechanisms of denitrifier as responses to biochar amendment.

Applicable schematic model for predication of N_2_O fluxes is necessary for estimating the GHGs emission under various biogeochemical parameters, and could offer potential implications for GHGs mitigation. Currently, most of the developed N_2_O models were associated with physicochemical characteristics (e.g., pH, water content, oxygen level, climatic information, nitrogen inputs, etc.) or potential denitrification/nitrification rates (Hu et al. 2015). However, limitations of these models in predication of N_2_O emissions in different circumstances have also been marked, and there is an urgent demand to exploit novel N_2_O emission models on account of nitrogen-cycling microbes or indicator genes determined by molecular strategies (Wallenstein and Hall 2011). In this study, the significant correlation between N_2_O fluxes and bacterial denitrification genes population, as well as *nirK*-*nosZ* value (Fig. 6a, b) was supported by previous studies (Wang et al. 2013). The derived linear regression model based on *nirK*-*nosZ* value could explain 80 % of the variance in N_2_O fluxes during windrow composting, which was similar to the model raised recently using *nirS*-*nosZ* value as the proxy (Morales et al. 2010). A previous field study also highlighted the linear correlation between *nirS*-*nosZ* value and N_2_O fluxes (Morales et al. 2010). It has been documented that nitrite reductase encoded by *nirS* was predominate as compared with *nirK* in most natural environments (Bothe et al. 2000), while it seemed that *nirK* gene was more dominant in composting systems (Wang et al. 2013). Furthermore, we developed a modified model for predicting of the N_2_O fluxes associated with the detailed abundances of denitrification functional genes (*nirK* and *nosZ*) during the manure composting (Fig. 7). The higher explaining fitting of this model (explaining 88 % of the variance in N_2_O fluxes) as compared with the linear model suggested that this linear equation was more effective in predicating N_2_O fluxes during composting based on the abundance of relevant functional genes (Fig. 7). This model highlighted the significant roles of denitrification in N_2_O emissions, which was the balance between N_2_O production (catalyzed by *nirK*) and consumption (catalyzed by *nosZ*) (Maeda et al. 2011; Wang et al. 2013).

Biochar amendment could also alleviate the N_2_O emissions through other ways independent of bacteria. For example, metal oxides, such as TiO_2_ distributing near the biochar, could catalyze the reduction of N_2_O to N_2_ (Ovideo and Sanz 2005). Moreover, the concentration of NO_3_^−^ was observed to be significantly lower in the biochar-amended piles than in the control piles, which could also have reduced N_2_O fluxes. However, whether the reduced NO_3_^−^ was directly attributed to the biochar amendment or indirectly caused by the effect of biochar on microbial metabolism still needs further investigation.

Besides of the mitigation effects on N_2_O emissions, biochar amendment also revealed other positive effects on manure composting. It was observed that addition of biochar accelerated the temperature rising and prolong the thermal stage of the composting (Fig. 3), which could improve the degradation of the organic substrates and shorten the composting period. This phenomenon has also been reported previously, and the involved mechanisms might be improvements of aeration and nutrients brought by the biochar (Wang et al. 2013).

In conclusion, this research presented that biochar amendment significantly reduced N_2_O emissions from manure composing, primarily through alternation of abundance of denitrification genes (*nirK*). Our study also highlighted the significant positive correlation between *nirK*-*nosZ* value and N_2_O fluxes, and developed a schematic model for predicting of the N_2_O fluxes associated with denitrification functional genes. It should be noticed that compared with advanced strategies such as next-generation sequencing and transcriptional profiling analysis, q-PCR has limitations both on data size and reliability (activity in vivo). Therefore, in the future more available approaches should be used for deeply exploring the microbial process involved in the biochar-mediated mitigation, especially in the detailed response of denitrification groups to biochar.
